# Ethical leadership and TMT decision-making of corporate social responsibility – a perspective of self-determination theory

**DOI:** 10.3389/fpsyg.2023.1268091

**Published:** 2023-12-08

**Authors:** Jia-jia Meng, Xue-dong Wang, Ming-yi Xie, Zhi-ling Hao, Jia-lu Yang, Yu-bing Liu

**Affiliations:** ^1^Sun Wah International Business School, Faculty of Economics, Liaoning University, Shenyang, China; ^2^School of Maritime Economics and Management, Dalian Maritime University, Dalian, China; ^3^Asia-Australia Business College, Liaoning University, Shenyang, China; ^4^School of Economics and Management, Dalian University of Technology, Dalian, China; ^5^Economics and Management School, Wuhan University, Wuhan, China

**Keywords:** ethical leadership, TMT harmonious work passion, TMT obsessive work passion, proactive CSR, reactive CSR

## Abstract

This study examines the impact of ethical leadership on top management team (TMT) decision-making regarding corporate social responsibility (CSR), considering the mediating role of TMT passion and the moderating role of performance stress. The study distinguishes between TMT harmonious and obsessive work passion and categorizes CSR as proactive and reactive. The findings reveal the following: (1) Ethical leadership positively influences proactive CSR, with TMT harmonious work passion acting as a positive mediator and TMT obsessive work passion playing a negative mediating role; (2) ethical leadership positively affects reactive CSR, with both TMT harmonious and obsessive work passion serving as positive mediators; (3) performance stress diminishes the impact of ethical leadership on TMT harmonious work passion; however, it amplifies the effect on TMT obsessive work passion. Consequently, the mediating effect of TMT harmonious work passion weakens, while the mediating effect of TMT obsessive work passion strengthens. This study emphasizes the significant role of TMT in CSR strategic decision-making and proposes a novel mediating mechanism through which ethical leadership drives CSR decision-making by considering TMT work passion. These findings reconcile the theoretical-practical conflict and have important theoretical and practical implications for enterprises in fulfilling their social responsibility.

## 1 Introduction

As a critical strategic decision-making, corporate social responsibility (CSR) has long been a focal topic in academic and practical fields (Fatima and Elbanna, 2023; Meng et al., 2023a). Although multilevel factors such as the institutional level (Li et al., 2019), organizational level (Luo and Wang, 2019), and individual level (Jiang et al., 2018) all influence CSR decision-making, leaders are considered to be the core internal antecedents (Pascricha et al., 2018). Moreover, among different stakeholders, the role of senior managers may be crucial as they are responsible for developing appropriate strategies, acquiring and utilizing necessary resources, and achieving organizational goals and performance in the right direction (Dey et al., 2022). The upper echelons theory suggests that corporate strategic decisions as a function of the mental characteristics of managers (Hambrick and Mason, 1984). As key decision-makers, CEO plays a crucial role (Sun et al., 2023) and CEO power also influences CSR activities (Kwon et al., 2023), therefore, research on the intersection of leadership and CSR is flourishing. Scholars have investigated the influence of leaders on CSR decision-making from the perspectives of CEOs’ observability characteristics (Shantaram and Mishra, 2019), psychological characteristics (Oh et al., 2019), and leadership (Silvestri and Veltri, 2019). Transformational leadership (Waldman et al., 2010), responsible leadership (Maak et al., 2016), servant leadership (Lythreatis et al., 2021), and ethical leadership (Musenze and Mayende, 2023) have been frequently mentioned. Among the diverse leadership styles, ethical leadership can more directly assess the ethical quality of leaders (Hoang et al., 2023) and hence has gradually become the most prominent leadership style that influences CSR strategic decision-making (Pascricha et al., 2018). Furthermore, existing research seems to reach such a consensus that ethical leadership has a direct positive impact on CSR decision-making (Saha et al., 2020). However, an anomaly that contradicts the general consensus in the literature is that “enterprises with the ethical CEO differ greatly in their CSR decision-making.” Those firms are in the same industry, have comparable sizes, and their leaders have distinct ethical leadership traits (Slavec Gomezel and Stritar, 2023). Meanwhile, some of them only fulfill their social responsibilities in a moderate manner according to the requirements of laws and regulations (Khan et al., 2023), while others go beyond the basic requirements and have taken the initiative to reconstruct the stakeholder landscape to achieve sustainable economic, social, and environmental development (Arvidsson and Dumay, 2022). The conflict between theory and practice raises the following question: “Why do similar ethical leaders make very different CSR decisions?”

The existing research on ethical leadership and CSR decision-making reveals several gaps, which together form an intriguing narrative. Firstly, while many studies have focused on the direct positive impact of ethical leadership on CSR decision-making, only a handful have delved into the mediating mechanisms from the perspectives of organizational culture and intellectual capital. These studies, conducted by Saha et al. (2020), Xuan et al. (2019), and Ullah et al. (2019), shed light on the importance of understanding the role of organizational culture, including organic, ethical, and moral culture, as well as intellectual capital, in the context of ethical leadership and CSR decision-making. However, a comprehensive understanding of this relationship remains limited. Secondly, the significant role of the top management team (TMT) in CSR decision-making has been largely overlooked in current studies. Despite the fact that organizational strategic decisions are not solely made by the CEO, but rather result from collective decision-making by the TMT, little attention has been given to exploring the influence of the TMT on CSR decision-making. Recognizing the importance of the TMT’s involvement in these decisions is essential for a comprehensive understanding of the dynamics at play. Finally, existing research has predominantly treated CSR as a single continuous variable, focusing primarily on changes in its “quantity” rather than exploring its “quality.” Lee (2011) has highlighted the need to investigate the different dimensions of CSR, distinguishing between proactive and reactive CSR initiatives. Such a distinction allows for a deeper exploration of the underlying motivations and strategies associated with CSR decision-making.

To address the existing gaps in the literature, the primary objective of this study is to shed light on the crucial role of top management teams (TMTs) in the decision-making process of corporate social responsibility (CSR). Moreover, this study aims to elucidate the underlying mechanism by which ethical leadership influences the heterogeneous nature of CSR decision-making.

Self-determination theory (SDT) provides insight from the theoretical perspective which builds a bridge between ethical leadership and different types of CSR decisions. Managers’ individual motivations include intrinsic motivation, which originates from intrinsic, and extrinsic motivation, which arises from rewards, punishments, and avoidance. As an external social norm imposed on TMT members, CSR can become a lasting strategic choice only after TMT members transform it from extrinsically motivated to intrinsically motivated (internalization of extrinsic motivation). According to the self-determination theory (Deci and Ryan, 1985a), the internalization process of extrinsic motivation may stimulate two types of work passion among TMT members: autonomous internalization generates harmonious work passion (HWP; Dong et al., 2011), and controlled internalization produces obsessive work passion (OWP), with different work passions leading to different CSR strategic choices (Luo et al., 2022). Therefore, TMT work passion forms a bridge between CEO ethical leadership and different types of CSR decision-making. In addition, TMT decision-making is always influenced by performance stress in the CSR strategic orientation that balances economic and social benefits (Mo et al., 2022). Performance stress perception affects the degree to which individuals satisfy their autonomy, competence, and relationship needs and the work passion derived from them (Deci and Ryan, 2000). It is inferred that performance stress may play a moderating role between ethical leadership and TMT work passion. In sum, self-determination theory provides a theoretical framework to explain how ethical leadership influences the intrinsic and extrinsic motivation of the TMT, thereby impacting corporate social responsibility decision-making.

Therefore, this study focuses on the question, “How does ethical leadership influence different types of CSR strategic decision-making?” Based on the self-determination theory, this study introduces TMT work passion as mediating variable as well as the performance stress as moderating variable, and adopts the empirical methodology to reveal the relationship between ethical leadership and CSR decision-making, thereby, enrich the research on the intersection of leadership and CSR fields.

## 2 Hypotheses development

### 2.1 Ethical leadership and CSR

Ethical leadership refers to “the demonstration of normatively appropriate conduct through personal actions and interpersonal relationships, and the promotion of such conduct to followers through two-way communication, reinforcement, and decision making” (Brown and Treviño, 2006; Pascricha et al., 2018). Ethical leader exemplifies the virtuous role model, exudes moral charisma and influences others by shaping an organization’s ethical culture through the process of ritualization (Yuan et al., 2023). CSR is defined as a company’s sense of responsibility towards the community and environment in which it operates, expressed by achieving economic, social, and environmental benefits considering the expectations of stakeholders (Carrol, 1979; Christensen et al., 2014). CSR can be categorized as philanthropic, integrative, and innovative by content (Halme and Laurila, 2009), reactive and proactive by attitude (Torugsa et al., 2013), and symbolic and substantive by form (Schons and Steinmeier, 2016). In these classifications, the more mainstream classification of reactive and proactive CSR is adopted in this study.

Ethical leadership is closely related to proactive CSR. Proactive CSR illustrates the “corporate integrity and ethical behavior that goes beyond the basic requirements of compliance to proactive initiatives such as innovation, eco-efficiency, and pollution prevention in support of sustainable economic, social, and environmental development” (Torugsa et al., 2013). On the one hand, proactive CSR depends on corporate discretion rather than reflecting governmental authority or broader institutional constraints (Matten and Moon, 2008). Therefore, only ethical leaders who are deeply committed to ethical concepts such as “honesty, integrity, trustworthiness, and caring” can act proactively beyond conventional legitimacy and integrate CSR into the decision-making process without only considering short-term economic interests. On the other hand, ethical leaders communicate their ethical values by establishing clear expectations for ethical behavior (Cheng et al., 2022); promote ethical consistency throughout the organization by cultivating the ethical values of organizational members (Kim et al., 2023); and create an ethical culture within the organization, therefore, encourage organizational members to take responsibility for ethical decisions, consider the sustainability of ethical decisions, put the interests of the organization and society above personal interests, and pay much attention to long-term interests instead of short-term interests (Khan et al., 2021). To sum up, we propose the following hypothesis:

*H1*: Ethical leadership has a positive effect on proactive CSR.

Ethical leadership is also closely related to reactive CSR. Reactive CSR refers to “enterprises passively make their business activities conform to the requirements of existing laws, regulations and procedures and meet the expectations of existing stakeholders in order to obtain and maintain the legitimacy of their operations under the external pressure of the institutional environment and market rules” (Torugsa et al., 2013). On the one hand, ethical leaders emphasize the “ethical individual,” as leaders, they advocate “normative behaviors” such as honesty, fairness, trustworthiness, and caring for others (Brown and Treviño, 2006; Nguyen et al., 2021), which suggests that they do not undermine ethical standards in the pursuit of short-term goals and can take into account the interests of existing stakeholders when pursuing the economic goals (Uppathampracha and Liu, 2022) and these continuous ethical behaviors enable the company to have a demonstration effect of “undertaking social responsibility.” On the other hand, ethical leaders emphasize “ethical others” (Verdorfer and Peus, 2019); that is, as leaders, they formulate and communicate ethical standards through two-way communication, continuous reinforcement, and decision-making and continuously promote ethical behavior among their followers (Brown and Treviño, 2006), while motivating the entire enterprise to comply with the basic requirements of laws and regulations in the course of conducting business activities, to take “social responsibility” in strategic decision-making, and to engage in business activities in a fair and principled manner (Nguyen et al., 2021). To sum up, we proposed:

*H2*: Ethical leadership has a positive effect on reactive CSR.

### 2.2 The mediating role of TMT harmonious work passion

Harmonious Work Passion (HWP) is “an autonomously internalized work passion of individuals who choose to engage in their favorite activities” which can play a mediating role between ethical leadership and both proactive and reactive CSR strategic decision-making.

Ethical leadership is conducive to the formation of TMT HWP. Ethical leaders with high level of “ethical ego,” their high ethical quality makes them more likely to be respected by TMT members who unconsciously generate HWP through autonomous internalization under the example and inspiration of ethical leaders (Gao and Jiang, 2019). Meanwhile, ethical leaders have distinctive characteristics of ethical communication in the process of communicating with TMT members, and their leadership style of treating people equally, caring for subordinates, and making fair decisions encourages a positive emotional state among TMT members (Al Halbusi et al., 2022), which stimulates TMT members’ emotional identification and motivates them to “choose an activity voluntarily and without any strings attached,” forming an autonomously internalized HWP (Jan et al., 2021). Based on the above arguments, we proposed that:

*H3*: Ethical leadership has a positive effect on TMT harmonious work passion.

TMT harmonious work passion facilitates the formation of both proactive and reactive CSR. Harmonious work passion is an autonomously internalized work passion composed of emotional and cognitive elements, which makes it easier for TMT members to accept CSR strategic decisions according to the business operations (Yi et al., 2022). Both reactive and proactive CSR require enterprises to assume social responsibility in addition to economic responsibility, which is a special strategic decision and an “additional” behavior for business organizations in general (Carrol, 1979). In terms of emotion, HWP allows TMT members to form individual commitment to and the will to take action on social responsibility “without any strings attached” (Vallerand et al., 2010) to develop a strong tendency and willingness to carry out CSR activities, and to implement CSR activities autonomously and voluntarily (Martin et al., 2022). In terms of cognition, HWP makes TMT members realize the importance of CSR to the sustainable development of enterprises and make CSR strategy decisions flexibly according to the enterprise’s particular situation. In other words, TMT HWP may lead enterprises to make both reactive and proactive CSR strategic decisions. Based on the above arguments, we propose the following hypothesis:

*H4*a: TMT harmonious work passion has a positive effect on proactive CSR.

*H4*b: TMT harmonious work passion has a positive effect on reactive CSR.

In summary, under the guidance and inspiration of ethical leaders, TMT members generate harmonious work passion through autonomous internalization. Harmonious work passion represents the positive side of work passion triggered by ethical leadership, accompanied by positive and adaptive results, allowing TMT members to willingly and flexibly carry out CSR strategic decision-making, which is conducive not only to reactive CSR formation but also to proactive CSR decision-making. To sum up, we propose the following hypothesis:

*H5*a: TMT harmonious work passion mediated the relationship between ethical leadership and proactive CSR.

*H5*b: TMT harmonious work passion mediated the relationship between ethical leadership and reactive CSR.

### 2.3 The mediating role of TMT obsessive work passion

Obsessive work passion (OWP) refers to “the internalization of an activity as part of the self-identity of an individual forced by external pressure, and it causes the individual to be compelled to engage in the activity out of a sense of inner compulsion.” Obsessive work passion plays different mediating roles between ethical leadership and different types of CSR strategic decision-making.

Ethical leadership may also cause TMT members to form an obsessive work passion. Compared to legal norms such as compliance with laws and regulations, ethics is a red-line behavior beyond the legal bottom line (Wu et al., 2015). Unlike law, which uses right and wrong as the standard, ethicality judge things with good and evil, which is a kind of value judgment that is difficult to measure effectively. According to self-determination theory, different people make different judgments when faced with same situation (Deci and Ryan, 1985b). In the presence of a strong ethical benchmark by ethical leaders, TMT members may be inspired to form an HWP by autonomous internalization or form an obsessive work passion by controlled internalization. Ethical leaders practice values-based management by strictly communicating ethical standards to their followers (Amah, 2023), providing clear ethical policies and programs, communicating about ethical issues, and using reinforcement systems to hold followers accountable for their ethical decisions (Pascricha et al., 2018). Therefore, TMT members feel the ubiquitous ethical oppression of ethical leaders and are thus “forced” to engage in an activity (Fu et al., 2020), forming an obsessive work passion by controlled internalization. Thus, we proposed as follows:

*H6*: Ethical leadership has a positive effect on TMT obsessive work passion.

TMT obsessive work passion is conducive to reactive CSR decision-making but not to proactive CSR decision-making. As a kind of work passion formed by controlled internalization, obsessive work passion only leads TMT members to engage in CSR activities in a controlled manner. The starting point for CSR decision-making by TMT members is more under the strong ethical pressure from ethical leadership (Sanchez-Famoso et al., 2023), so it becomes a natural strategic choice for TMT members to engage in some superficial work to “cope” (Mageau et al., 2011). The reactive CSR strategic decisions that “conform corporate behavior to the basic requirements of laws and regulations, cater to existing stakeholders, and enhance the legitimacy of the company” become the natural choice of TMT members.

Meanwhile, the obsessive work passion formed through controlled internalization simply fails to create positive emotional outcomes among TMT members, and proactive CSR strategic decisions that “go beyond compliance, proactively reconstruct stakeholders, and support social sustainability” are hardly preferred by TMT members in the context of obsessive work passion (Vallerand et al., 2010). In particular, with big group of TMT members, the limited decision-making power of ethical leaders makes it difficult to implement proactive CSR strategic decisions as a collective choice of all TMT members in the face of many TMT members with obsessive work passion (Zolotoy et al., 2021). Under such circumstances, enterprises are more inclined to engage in reactive CSR, and it is difficult for them to adopt and implement proactive CSR strategic decisions. Thus, we proposed:

*H7*a: TMT OWP has a negative impact on proactive CSR.

*H7*b: TMT OWP has a positive effect on reactive CSR.

In sum, TMT members may develop obsessive work passion under the ethical pressure from ethical leadership. Obsessive work passion represents the negative side of work passion triggered by ethical leadership, accompanied by passive, adaptive outcomes (Nie et al., 2023). It is not the TMT’s job to take social responsibility beyond the rules (Lafrenière et al., 2011), while social responsibility within the rules is the norm must be followed. TMT members under obsessive work passion can only make reactive CSR decisions, and it is difficult for them to make proactive CSR decisions. Therefore, the following hypotheses are proposed:

*H8*a: TMT obsessive work passion mediated the relationship between ethical leadership and proactive CSR.

*H8*b: TMT obsessive work passion mediated the relationship between ethical leadership and reactive CSR.

### 2.4 The moderating role of performance stress

Economic responsibility is the primary issue of CEO and TMT members (Cheong et al., 2017). In a market-oriented business organization, CSR is always faced with the consideration of economic responsibility, and the direct result of economic responsibility is corporate performance (Qu et al., 2022). Therefore, the performance stress faced by TMTs becomes an important situational factor that affects TMT work passion. Performance stress refers to “the sense of urgency to improve corporate performance to achieve desired outcomes and avoid negative consequences” (Lazarus, 2000). Performance stress plays different moderating roles in the relationship of ethical leadership with different work passion of TMT.

Performance stress positively moderated the relationship between ethical leadership and different TMT work passion. According to the theory of resource conservation (Luan et al., 2022), the signals of resource changes received by individuals significantly affect their subsequent attitudes and behaviors (Hobfoll, 1989). Therefore, when TMT members are faced with large, realistic, and urgent performance stress, they develop a significant sense of being threatened or hurt, which in turn affects their subsequent emotional responses (Folkman and Lazarus, 1985). The greater the performance stress, the higher the self-protection needs of TMT members, which are concentrated in their perception of self-service (Mitchell et al., 2017). Therefore, under the example and inspiration of ethical leaders, it is more likely that control internalization will cause TMT members to develop obsessive work passion. Moreover, the ethical enlightenment and inspiration of ethical leaders appear feeble and futile in the face of realistic performance stress: in the face of performance stress that is closely related to their own positions and pay incentives (Linder et al., 2021), it is almost impossible for TMT members to form autonomously internalized harmonious work passion with heartfelt pleasure. Therefore, we proposed:

*H9*a: Performance stress negatively moderated the relationship between ethical leadership and TMT harmonious work passion; that is, the higher the performance stress is, the weaker the positive effect of ethical leadership on TMT harmonious work passion.

*H9*b: Performance stress positively moderated the relationship between ethical leadership and TMT obsessive work passion; that is, the higher the performance stress is, the stronger the positive effect of ethical leadership on TMT obsessive work passion.

In sum, we propose a moderated mediation model for CSR decision making: CEO ethical leadership is related to CSR decision making by enhancing the work passion of TMT. Yet, a low level of performance stress is proposed to form a prerequisite for ethical leadership to translate into CSR decision making via the TMT harmonious work passion. In contrast, under the boundary condition of a high level of performance stress, it’s more likely to transfer ethical leadership to CSR decision making through TMT obsessive work passion. Thus, we propose the following hypotheses:

*H10*a: Performance stress moderates the mediating role of TMT HWP between ethical leadership and proactive CSR; that is, the higher the performance stress is, the weaker the mediating role of HWP.

*H10*b: Performance stress moderates the mediating role of TMT HWP between ethical leadership and reactive CSR; that is, the higher the performance stress is, the weaker the mediating role of HWP.

*H10*c: Performance stress moderates the mediating role of TMT OWP between ethical leadership and proactive CSR; that is, the higher the performance stress is, the stronger the mediating role of OWP.

*H10*d: Performance stress moderates the mediating role of TMT OWP between ethical leadership and reactive CSR; that is, the higher the performance stress is, the stronger the mediating role of OWP.

**Figure 1:**
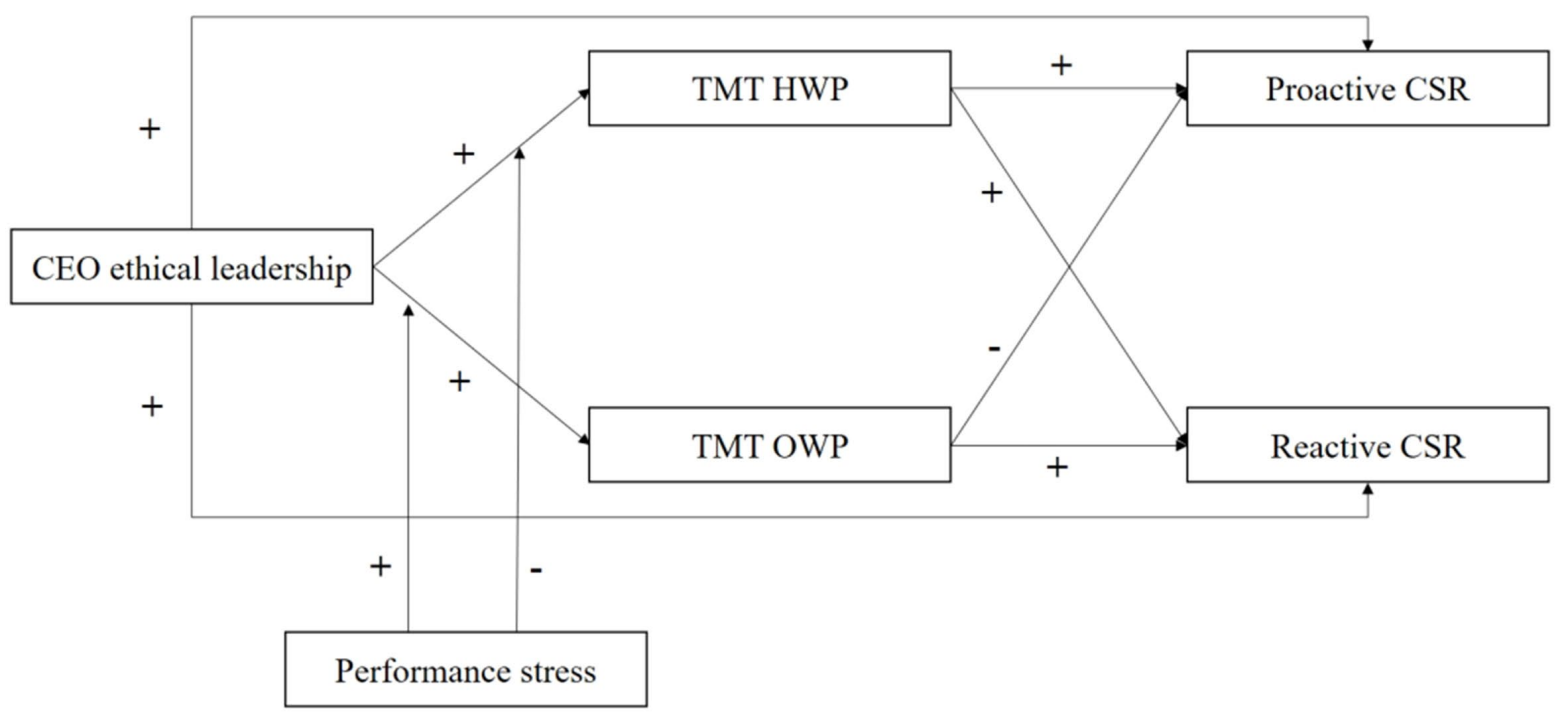
The theoretical model.

Based on the above hypotheses, the theoretical model proposed in this study is shown in Figure 1.

## 3 Materials and methods

### 3.1 Sample and data collection

The survey data was collected from a questionnaire within three-month. After selecting appropriate scales based on existing literature and incorporating expert opinions, we developed an initial survey questionnaire. To ensure the quality of the questionnaire, we distributed the initial questionnaire to a small group of executives in various companies. Based on their feedback, we further refined and improved the questionnaire, resulting in the final version used for the formal survey. We invited TMT members from six companies (four state-owned and two private enterprises) to participate in the initial questionnaire survey. However, these six companies were not included in the formal research sample. We considered their feedback and incorporated suggestions from domain experts to make appropriate modifications to the wording of the questionnaire items, ultimately creating the final version for the formal survey.

Participants for this study were recruited from Leaders and TMT members of the sample enterprises. The sampling technique employed for this study was convenience sampling. The selection of sample enterprises was mainly based on the following criteria: (1) the sample enterprises regularly publish CSR reports; (2) the sample enterprises have a complete TMT; and (3) the data of the sample enterprises were complete and available. The data were collected using the CEO–other TMT members pairing method. The questionnaire was divided into parts A and B. The CEO completed part A of the questionnaire to evaluate basic personal information, basic corporate information, proactive CSR, and reactive CSR. Four to five other TMT members were randomly selected to fill out part B of the questionnaire to evaluate ethical leadership, HWP, OWP, and performance stress. After the elimination of incomplete, duplicate, and invalidly paired questionnaires and those showing patterned responses, 506 valid questionnaires (including 122 questionnaires in part A and 506 questionnaires in part B) were obtained. The structural characteristics of CEOs and sample are presented in Table 1.

**Table 1:** Structural characteristics of corporate CEOs and sample enterprises.

Structural characteristics of survey subjects				Structural characteristics of the sample enterprises			
Variable	Category	Number of people	Proportion	Variable	Category	Number of enterprises	Proportion
CEO gender	Male	113	92.62%	Type of enterprise	Manufacturing industry	54	44.26%
	Female	9	7.38%		Construction industry	13	10.66%
CEO age	30 years old or younger	5	4.10%		Information technology industry	23	18.85%
	31–40 years old	19	15.57%		Transportation industry	17	13.93%
	41–50 years old	53	43.44%		Other	15	12.30%
	51–60 years old	35	28.69%	Type of ownership	State-owned enterprise	43	35.25%
	60 years old or more	10	8.20%		Private enterprise	58	47.54%
CEO education level	Bachelor	82	67.21%		Other	21	17.21%
	Masters’	38	31.15%	Location	Eastern region	34	27.87%
	Doctorate	2	1.64%		Northeastern region	49	40.16%
					Central region	29	23.77%
					Western region	10	8.20%

### 3.2 Measures

To avoid connotation inconsistency, the scales in this study were all obtained from published English literature and were processed using a strict translation/backtranslation procedure. Except for the control variables, the questionnaire items were measured on a five-point Likert scale ranging from 1 (strongly disagree) to 5 (strongly agree).

#### 3.2.1 Ethical leadership

We measured ethical leadership by adopting a ten-item scale from Brown and Treviño (2006). Sample item: “The CEO of our company makes fair and balanced decisions.”

#### 3.2.2 Proactive CSR and reactive CSR

Proactive CSR and reactive CSR were measured using a 5-item scale individually which all developed by Chang (2015). Sample item: “Our company passively carries out public service activities to meet social expectations.”

#### 3.2.3 HWP and OWP

HWP and OWP were assessed by adapting a seven-item scale from Vallerand et al. (2010). Sample item: “I am completely happy with my current work” and an example item for OWP being “I can hardly imagine my life without my current work.”

#### 3.2.4 Performance stress

Performance stress was assessed by using a four-item scale from Mitchell et al. (2017). Sample item: “I feel that my job will be at risk if I do not maintain a high level of performance.”

In addition, based on existing studies (Brik et al., 2011; Wu et al., 2015), the type of enterprise, type of ownership, and location were used as control variables.

## 4 Results

### 4.1 Aggregation test

For the measurement of high-level constructs in this study comes from individuals and are finally obtained through the aggregation of individual data. However, the premise of the aggregation is to ensure that the consistency of the views of team members reaches a certain standard, therefore, aggregation test was used to assess the consistency (James, 1982; Chan, 1998).

Rwg, ICC (l), and ICC (2) are statistics generally used to verify aggregation of data to higher levels of analysis. As shown in Table 2, the mean values of Rwg for each variable are ranged from 0.87 to 0.89, as well as the median values of Rwg are between 0.86 to 0.90, which all greater than the suggested value of 0.70 from LeBreton and Senter (2008). Beyond, ICC (1) for each variable is varied from 0.27 to 0.55, which is greater than the recommended cutoff of 0.12 from LeBreton and Senter (2008). In addition, although there are no strict standards of acceptability for ICC (2) values, Glick (1985) reported an ICC (2) value of 0.60 in the organizational literature. The Values of ICC (2) in this study are ranged from 0.58 to 0,84, most of the values are >0.60, which only one is slightly below the recommended level, it does not seem low enough to prohibit aggregation considering the small size of the tested team. In sum, the above results provided sufficient support for aggregation.

**Table 2:** Results of aggregation test.

Variable	Rwg mean	Rwg median	ICC (1)	ICC (2)
CEO ethical leadership	0.89	0.90	0.27	0.61
TMT HWP	0.87	0.86	0.25	0.58
TMT OWP	0.88	0.89	0.41	0.74
Performance stress	0.87	0.88	0.55	0.84

### 4.2 Reliability and validity test and correlation analysis

#### 4.2.1 Reliability and validity test

Established scales are used in this study, and the results of reliability and validity test were shown in Table 3. Cronbach *α* of all variables range from 0.778 to 0.963 and CR values range from 0.832 to 0.963, which are all >0.7, therefore, demonstrating that the scales reach to high reliability. All factor loadings are >0.60, and values of average variance extracted (AVE) are between 0.416 to 0.836, which are almost >0.50. In general, AVE above 0.5 or with a CR value above 0.7 indicates higher convergent validity. Therefore, although the AVE value of Reactive CSR is slightly less than 0.5, its CR value is 0.780, which can also be accepted.

**Table 3:** Results of reliability and validity test.

Variable	Cronbach’s α	CR	Factor loading	AVE
1. CEO ethical leadership	0.963	0.963	0.817 ~ 0.891	0.725
2. TMT HWP	0.945	0.946	0.824 ~ 0.883	0.713
3. TMT OWP	0.956	0.956	0.839 ~ 0.900	0.756
4. Proactive CSR	0.830	0.832	0.634 ~ 0.801	0.500
5. Reactive CSR	0.778	0.780	0.605 ~ 0.693	0.416
6. Performance stress	0.953	0.953	0.903 ~ 0.924	0.836

Additionally, confirmatory factor analysis (CFA) was used to evaluate the measurement model, and the results show that the goodness of fit of the six-factor model is acceptable (*χ^2^/df =* 1.139 < 3, IFI = 0.976 > 0.9, TLI = 0.974 > 0.9, CFI = 0.976 > 0.9, and RMSEA = 0.034 < 0.08), indicating a good discriminant validity of the scale (Fornell and Larcker, 1981).

#### 4.2.2 Common method bias test

In this study, Harman’s one-factor analysis was used to test the common method bias. After factor analysis of the variables of CEO ethical leadership, TMT HWP, TMT OWP, proactive CSR, reactive CSR, and performance stress, the cumulative explained variance in the un-rotated first factor was 30.118%, less than the critical value of 40.0%. Moreover, the CFA results show that the six-factor model has a good fit, while the one-factor model does not reach (and is, in fact, far from) the standard range for each indicator, i.e., it has a poor fit, indicating that the constructs can be distinguished from each other. The above methods indicate that there is no significant common method bias in this study.

#### 4.2.3 Correlation analysis

Descriptive statistics and correlation analysis of the study variables were conducted using SPSS 26.0, and the results are presented in Table 4. According to the correlation coefficients, ethical leadership is significantly positive related with proactive CSR (*r* = 0.293, *p* < 0.01), reactive CSR (*r* = 0.397, *p* < 0.01), TMT HWP (*r* = 0.399, *p* < 0.01), and TMT OWP (*r* = 0.297, *p* < 0.01); TMT HWP is significantly positively correlated with proactive CSR (*r* = 0.385, *p* < 0.01) and reactive CSR (*r* = 0.440, *p* < 0.01); TMT OWP is significantly negative related with proactive CSR (*r* = −0.241, *p* < 0.01) and significantly positive related with reactive CSR (*r* = 0.456, *p* < 0.01). The above results provide preliminary support for hypothesis testing.

**Table 4:** Descriptive statistics and correlation analysis of main variables.

Variable	1	2	3	4	5	6
1. CEO ethical leadership	(0.851)					
2. TMT HWP	0.399^**^	(0.844)				
3. TMT OWP	0.297^**^	0.189^*^	(0.869)			
4. Proactive CSR	0.293^**^	0.385^**^	−0.241^**^	(0.707)		
5. Reactive CSR	0.397^**^	0.440^**^	0.456^**^	0.102	(0.645)	
6. Performance stress	−0.004	−0.218^*^	0.159	−0.236^**^	−0.126	(0.914)
Mean	3.394	3.278	3.130	3.592	3.608	2.868
Standard deviation	0.598	0.563	0.605	0.788	0.687	0.720

*N* = 122; the diagonal values in parentheses are square roots of AVEs for each variable. ^*^p < 0.05. ^**^p < 0.01, *p* < 0.001 (2-tailed), the same below.

### 4.3 Hypothesis testing

#### 4.3.1 Test of main and mediating effects

Bootstrap method in the PROCESS macro (Preacher and Hayes, 2008) was used to test all hypotheses of the main and mediating effects (i.e., *H1* to *H12*). The Bootstrap method is a statistical technique utilized to estimate the distribution of a statistic and conduct hypothesis testing through simulating repeated sampling. It was employed to test mediation effects and estimate confidence intervals for indirect effects in this study mainly based on the following reasons. Firstly, the Bootstrap method offers more flexibility regarding data distribution requirements, enabling a more robust and universally applicable mediation effect test without specific assumptions about the data distribution (Meng et al., 2023b). Moreover, in comparison to traditional analytical methods, the Bootstrap method imposes lower sample size requirements. This is particularly advantageous in small sample studies, where the Bootstrap method can yield more reliable results. Lastly, mediation effect tests often rely on estimated parameters such as regression coefficients. These parameter estimates can be influenced by factors like sample size and data skewness, potentially leading to inaccuracies in the test. By performing bootstrap resampling with replacement on the original data, the Bootstrap method generates a substantial number of simulated samples, helping to mitigate such errors and enhance the statistical robustness of the analysis.

As shown in Table 5, the regression results show that CEO ethical leadership is significantly positively correlated with proactive CSR (*β* = 0.353, *p* < 0.01) and reactive CSR (*β* = 0.450, *p* < 0.001), and thus *H1* and *H2* are supported.

**Table 5:** Bootstrap test results.

IV	M	DV	Effect of IV on M	Effect of M on DV	Total effect	Direct effect	Indirect effect	95% CI
EL	HP	PCSR	0.377***	0.457***	0.353**	0.180	0.172	[0.059, 0.313]
		RCSR		0.410***	0.450***	0.296**	0.155	[0.047, 0.313]
EL	OP	PCSR	0.315***	−0.435***	0.353**	0.490***	−0.137	[−0.241, −0.044]
		RCSR		0.420***	0.450***	0.318**	0.132	[0.037, 0.246]

Bootstrap results for multiple mediator tests								
Specific indirect effect			Total indirect effect		Direct effect		Total effect	IV
M	Effect	95% CI	Effect	95% CI	Effect	95% CI		
HP	0.187	[0.075, 0.320]	0.039	[−0.130, 0.211]	0.313	[0.008, 0.085]	0.353**	PCSR
OP	−0.148	[−0.267, −0.049]						
HP	0.142	[0.047, 0.287]	0.267	[0.144, 0.404]	0.184	[0.063, −0.010]	0.450***	RCSR
OP	0.124	[0.036, 0.222]						

##### 4.3.1.1 CEO ethical leadership, TMT HWP, and proactive (reactive) CSR

Hypotheses 3 was tested by adding CEO ethical leadership, TMT HWP, and proactive (reactive) CSR to the Process macro simultaneously (Model 4, with the two dependent variables entered separately). Table 5 shows that CEO ethical leadership is significantly positively correlated with TMT HWP (*β* = 0.377, *p* < 0.001). Thus, hypothesis 3 was supported. The indirect relationship between CEO ethical leadership on proactive CSR (*β* = 0.172, 95% CI = [0.059, 0.313]) and reactive CSR (*β* = 0.155, 95% CI = [0.047, 0.313]) through TMT HWP is significant, showing that TMT HWP mediates the positive relationship between CEO ethical leadership on proactive CSR and reactive CSR. Thus, hypothesis 5a and hypothesis 5b were supported. Meanwhile, the direct effect of CEO ethical leadership on proactive CSR is not significant (*β* = 0.180, *ns*), suggesting that TMT HWP shows “indirect-only mediation” (Zhao et al., 2010) between the two; the direct effect of CEO ethical leadership on reactive CSR is significant (*β* = 0.296, *p* < 0.01), indicating that TMT HWP is manifested as “complementary mediation” (Zhao et al., 2010) between the two.

##### 4.3.1.2 CEO ethical leadership, TMT OWP, and proactive (reactive) CSR

Hypotheses 6 was tested by adding CEO ethical leadership, TMT OWP, and proactive (reactive) CSR to the Process macro simultaneously (Model 4, with the two dependent variables entered separately). Table 5 shows that CEO ethical leadership is significantly positively correlated with TMT OWP (*β* = 0.315, *p* < 0.001). Thus, hypothesis 6 was supported. TMT OWP is significantly negatively correlated with proactive CSR (*β* = −0.435, *p* < 0.001) and significantly positively correlated with reactive CSR (*β* = 0.420, *p* < 0.001), supporting *H7*a and *H7*b. There are significant indirect effects of CEO ethical leadership on proactive CSR (*β* = −0.137, 95% CI = [−0.241, −0.044]) and reactive CSR (*β* = 0.132, 95% CI = [0.037, 0.246]) through TMT OWP, so *H8*a and *H8*b are supported. Meanwhile, the direct effect of ethical leadership on proactive CSR is significant (*β* = 0.490, *p* < 0.001), indicating that TMT OWP is manifested as “competitive mediation” (Zhao et al., 2010) between the two; the direct effect of CEO ethical leadership on reactive CSR remains significant (*β* = 0.318, *p* < 0.01), suggesting that TMT OWP shows “complementary mediation” (Zhao et al., 2010) between the two variables.

##### 4.3.1.3 Test of dual mediating effects

CEO ethical leadership, TMT OWP, TMT HWP, and proactive (reactive) CSR were entered into the PROCESS macro simultaneously (Model 4, with the two dependent variables entered separately). As shown in Table 5, there are significant specific indirect effects of CEO ethical leadership having a positive impact on proactive CSR through TMT HWP (*β* = 0.187, 95% CI = [0.075, 0.320]) and a negative impact on proactive CSR through TMT OWP (*β* = −0.148, 95% CI = [−0.267, −0.049]). Thus, hypothesis 5a and hypothesis 8a were supported. Furthermore, the specific indirect effects of ethical leadership having a positive impact on reactive CSR through TMT HWP (*β* = 0.142, 95% CI = [0.047, 0.287]) and through TMT OWP (*β* = 0.124, 95% CI = [0.036, 0.222]), are both significant; hence, *H5*b and *H8*b are further supported.

#### 4.3.2 Test of moderating effects

The moderating effects were tested using hierarchical regression, while the variables were decentralized to reduce the multi-collinearity. The test results are depicted in Table 6.

**Table 6:** Test results of moderating effects.

Variable	TMT HWP			TMT OWP		
	M1	M2	M3	M4	M5	M6
Industry type	0.029	0.011	0.022	0.039	0.022	0.013
Nature of ownership	−0.046	−0.007	−0.007	0.067	0.116	0.116
Location	0.022	0.034	0.047	0.013	0.025	0.013
Ethical leadership (EL)		0.374***	0.392***		0.318**	0.302**
Performance stress (PS)		−0.169*	−0.191**		0.142	0.161*
EL × PS			−0.283**			0.254*
*R^2^*	0.009	0.210	0.262	0.017	0.138	0.175
Δ*R^2^*	0.009	0.200**	0.053**	0.017	0.121***	0.037*
*F*	0.363	6.151***	6.812***	0.665	3.718**	4.056**

In Model 3, the interaction term between ethical leadership and performance stress has a significant negative impact on TMT HWP (*β* = −0.283, *p* < 0.01), indicating that performance stress negatively moderates the impact of ethical leadership on TMT HWP, so *H9*a is supported. In Model 6, the interaction term between ethical leadership and performance stress has a significant positive impact on TMT OWP (*β* = 0.254, *p* < 0.05), indicating that performance stress positively moderates the impact of moral leadership on TMT OWP; hence, *H9*b is verified.

**Figure 2:**
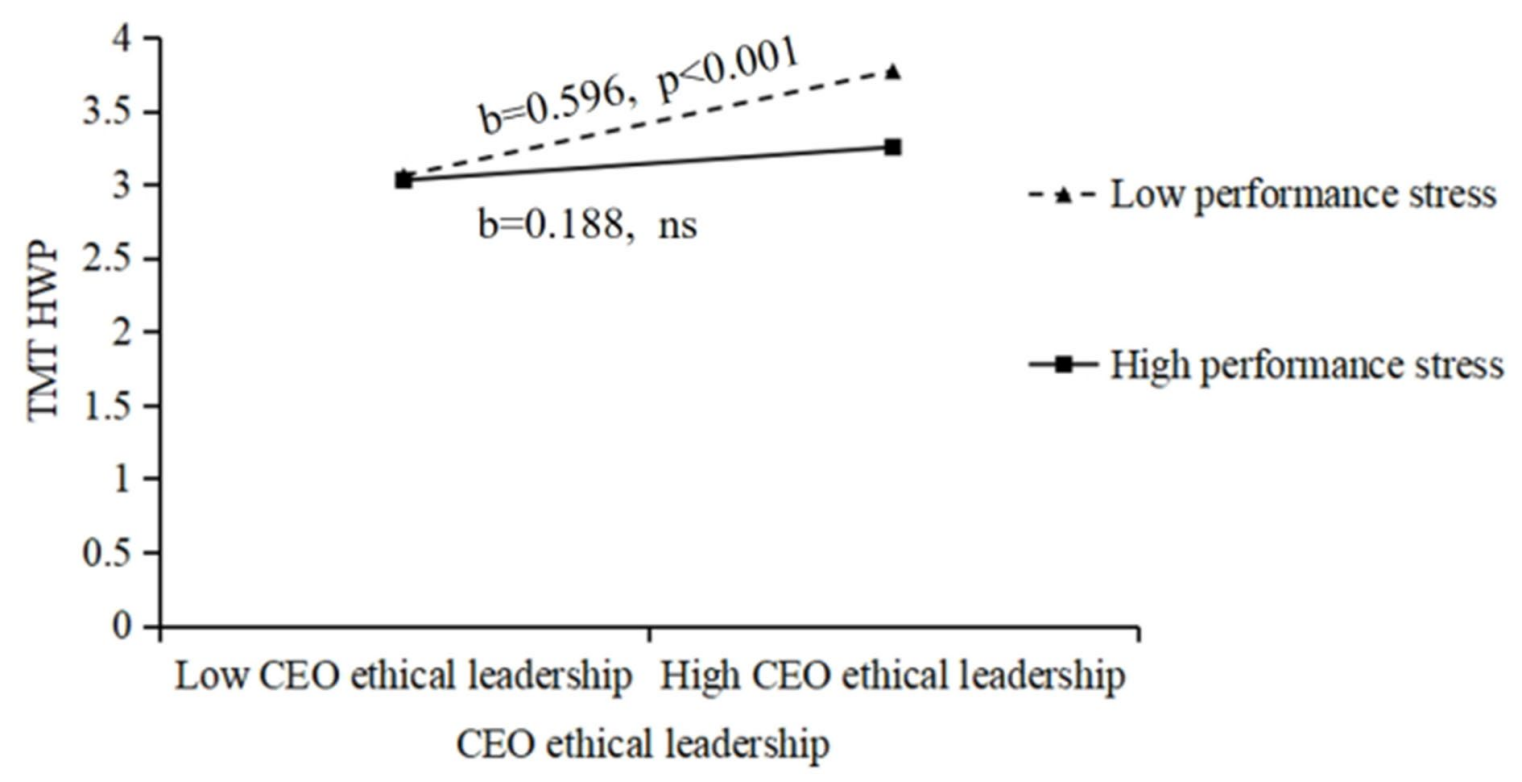
The moderating role of performance stress in the relationship between CEO ethical leadership and TMT HWP.

**Figure 3:**
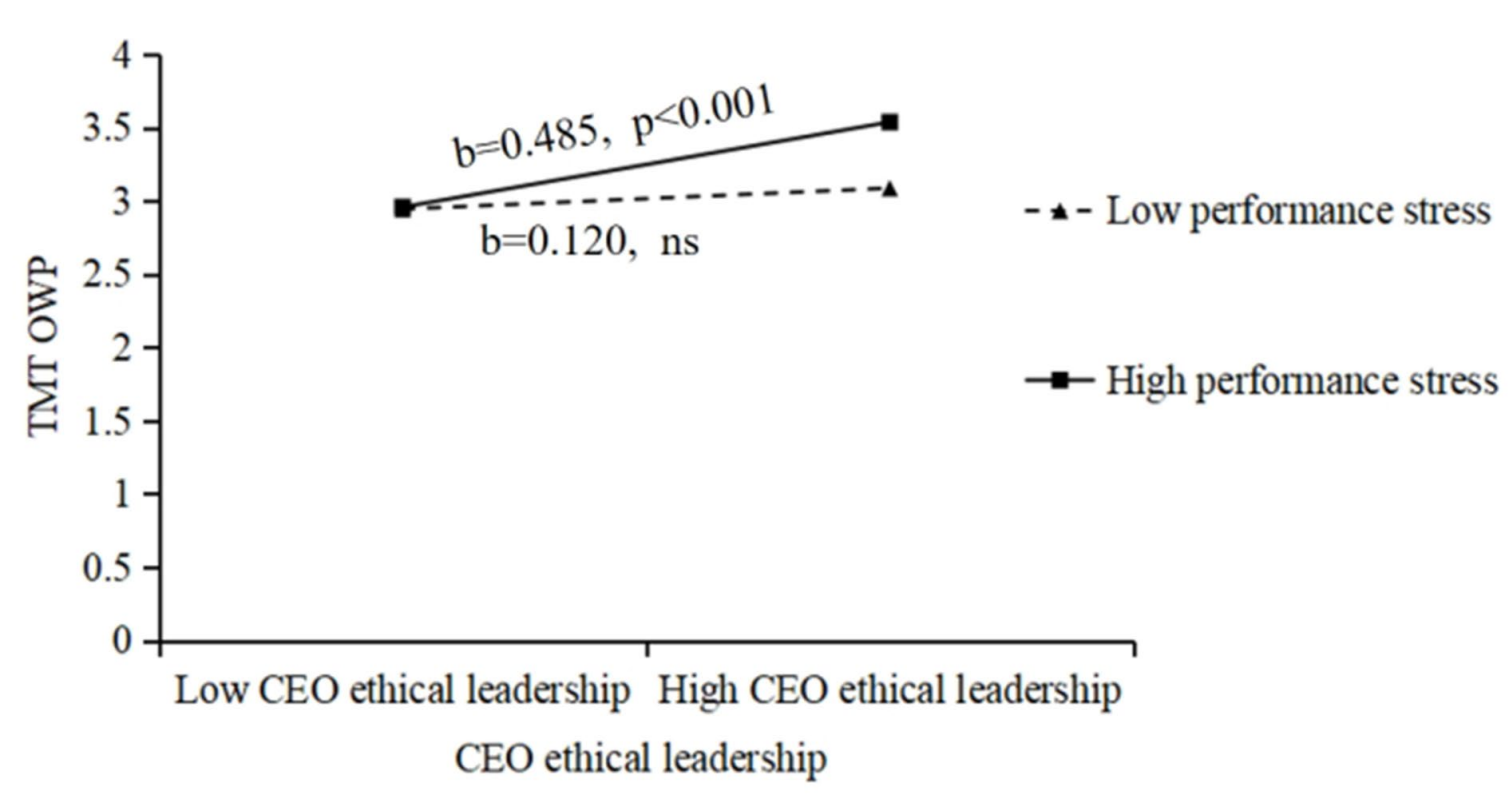
The moderating role of performance stress in the relationship between CEO ethical leadership and TMT OWP.

Diagrams of the moderating effects of performance stress are depicted for further visualization. In Figure 2, when performance stress is high, the relationship curve of ethical leadership and TMT HWP has a gentler simple slope and the effect is not significant (*b* = 0.188, *ns*), indicating a weaker positive impact of ethical leadership on TMT HWP; that is, performance stress negatively moderates the relationship between the two. In Figure 3, when performance stress is high, the relationship curve of ethical leadership and TMT OWP has a steeper simple slope (*b* = 0.485, *p* < 0.001), suggesting a stronger positive impact of ethical leadership on TMT OWP; that is, performance stress positively moderates the relationship between the two.

#### 4.3.3 Test of moderated mediating effects

The bootstrap test was performed using the SPSS PROCESS macro to verify the moderated mediating effects. The test results are presented in Table 7.

**Table 7:** Test results of moderated mediating effects.

Dependent variable	Mediating variable	Performance stress	Effect size	Standard error	95% CI	
					LLCI	ULCI
Proactive CSR	TMT HWP	Low	0.296	0.093	0.141	0.497
		High	0.093	0.072	−0.032	0.257
		Difference	−0.203	0.108	−0.430	−0.008
	TMT OWP	Low	−0.056	0.063	−0.174	0.081
		High	−0.228	0.079	−0.401	−0.093
		Difference	−0.172	0.095	−0.379	−0.009
Reactive CSR	TMT HWP	Low	0.225	0.074	0.091	0.384
		High	0.071	0.069	−0.024	0.246
		Difference	−0.154	0.071	−0.292	−0.005
	TMT OWP	Low	0.047	0.054	−0.084	0.139
		High	0.191	0.076	0.056	0.349
		Difference	0.144	0.091	0.008	0.356

As shown in Table 7, the difference in the mediating effect of TMT HWP through which ethical leadership promotes proactive CSR at high and low performance stresses is significant (*β* = −0.203, 95% CI = [−0.430, −0.008]), and the higher the performance stress, the weaker the mediating effect of TMT HWP, so *H10*a holds. The difference in the mediating effect of TMT HWP through which ethical leadership promotes reactive CSR at high and low performance stresses is also significant (*β* = −0.154, 95% CI = [−0.292, −0.005]), and the higher the performance stress is, the weaker the mediating effect of TMT HWP; hence, *H10*b is supported. The difference in the mediating effect of TMT OWP through which ethical leadership inhibits proactive CSR at high and low performance stresses is significant (*β* = −0.172, 95% CI = [−0.379, −0.009]), and the higher the performance stress, the stronger the mediating effect of TMT OWP, so *H10*c holds. Finally, the difference in the mediating effect of TMT OWP through which ethical leadership promotes reactive CSR at two different levels of the moderator (high and low performance stresses) is significant (β = 0.144, 95% CI = [0.008, 0.356]), and the higher the performance stress is, the stronger the mediating effect of TMT OWP; hence, *H10*d is verified.

## 5 Discussion and conclusion

This study aims to answer the following question: “What and how does CEO ethical leadership influence CSR strategic decision-making?” By introducing TMT HWP and TMT OWP as mediating variables and performance stress as a moderating variable, we construct a model of the mechanism by which CEO ethical leadership influences CSR strategic decision-making via the TMT and draw the following conclusions.

Firstly, regarding ethical leadership and proactive CSR strategic decision-making, ethical leadership has a positive impact on proactive CSR. In particular, TMT HWP is manifested as “indirect-only mediation” in the relationship between ethical leadership and proactive CSR decision-making; that is, ethical leadership can promote proactive CSR strategic decision-making only by enhancing TMT HWP. TMT OWP is manifested as “competitive mediation”; that means ethical leadership hinders proactive CSR decision-making by improving TMT OWP.

Secondly, regarding ethical leadership and reactive CSR strategic decision-making, ethical leadership has a positive impact on reactive CSR, and both TMT HWP and TMT OWP are manifested as “complementary mediation” in the relationship between ethical leadership and reactive CSR; that is, ethical leadership can drive reactive CSR strategic decision-making through both TMT HWP and TMT OWP.

Finally, regarding the moderating effects of performance stress, performance stress has negative and positive moderating effects between ethical leadership and TMT HWP and between ethical leadership and TMT OWP, respectively. Meanwhile, performance stress moderates the mediating effect of TMT work passion on the relationship between ethical leadership and proactive and reactive CSR; the higher the performance stress is, the weaker the mediating effect of TMT HWP and the stronger the mediating effect of TMT OWP.

Overall, our study provides an in-depth understanding of the relationship between ethical leadership, TMT, and CSR by comparing and contrasting with previous literature. These research findings have significant practical implications for organizational managers and decision-makers in formulating and implementing CSR strategies.

### 5.1 Theoretical contributions

Previously, scholars have emphasized the lack of research on CSR at the micro level (Aguinis and Glavas, 2013; Wu et al., 2015). However, the leadership literature offers a comprehensive process model and explanatory mechanism for understanding how individual leaders influence CSR. Similarly, the CSR literature provides leadership scholars with a focused background to investigate the influence of leaders. Therefore, combining these two areas of literature presents ample opportunities for theoretical development. This study aims to explore the impact of ethical leadership on CSR strategic decision-making, making a valuable attempt to examine the relationship between ethical leadership and CSR strategic decision-making. The main theoretical contributions of this study are as follows.

Firstly, this study draws on self-determination theory to propose a novel mediation mechanism that explains the intricate relationship between ethical leadership and heterogeneous CSR strategic decision-making from the perspective of the top management team (TMT) work passion. While previous studies have explored the relationship between ethical leadership and CSR, most have not provided an explanation for the underlying process, resulting in a lack of research on potential mediation mechanisms between ethical leadership and strategic CSR decision-making. By incorporating TMT harmonious work passion (HWP) and TMT obsessive work passion (OWP) as explanatory variables, this study examines how ethical leadership influences CSR strategic decision-making, integrating leadership, emotion, and behavior. Furthermore, it addresses the question of “how ethical leadership affects different types of CSR strategic decision-making,” enriching the understanding of the mediation mechanism between ethical leadership and CSR from an emotional perspective and providing a new process pathway to explain how ethical leadership differentially influences proactive and reactive CSR strategic decision-making.

Secondly, this study enhances our understanding of the significance of the TMT in CSR strategic decision-making and highlights its crucial role in the process. Previous studies on leadership and CSR have predominantly focused on CEOs, overlooking the TMT as an essential core group within organizations. By considering the TMT as a link between ethical leadership and CSR, this study reveals the internal mechanism of how ethical leadership influences heterogeneous CSR strategic decision-making within enterprises. It addresses the limitations of previous studies that primarily focused on CEO characteristics as the driving force behind CSR, contributing to expanding and deepening existing research. The TMT plays a primary role in designing and implementing stakeholder strategies such as CSR, and CSR practices are influenced by the philosophies and actions of not only the CEO but also the entire TMT. This study responds to the suggestions of Wu et al. (2015), Frynas and Yamahaki (2016), and Ahn (2020) to include the TMT in the explanation of CSR, revealing the TMT’s role in CSR strategic decision-making.

Thirdly, this study reconciles the conflicts between existing literature and corporate practices, refines the differentiated impact of ethical leadership on CSR, and challenges the stereotypical understanding of the positive impact of ethical leadership on CSR by conducting a heterogeneity analysis of CSR. Previous studies have primarily focused on general CSR activities and their determinants, with limited attention given to different types of CSR. This study addresses the call for further research on how managers prioritize and balance different types of CSR activities (Chen et al., 2019) by examining how CEO ethical leadership influences heterogeneous CSR strategic decision-making. By exploring proactive and reactive CSR in a categorical manner, it delves into the distinct generating mechanisms behind different CSR strategic decisions.

Overall, this study contributes to the literature by providing a comprehensive analysis of the relationship between ethical leadership and CSR strategic decision-making. By incorporating the TMT and exploring the mediating role of work passion, it offers new insights into the complex processes underlying this relationship. Additionally, it broadens our understanding of the TMT’s role in CSR decision-making and highlights the importance of considering diverse types of CSR.

### 5.2 Practical implications

This study has offer certain practical implications for corporate leaders, TMTs, and CSR strategic decision-making.

Firstly, leaders are the key influencers of CSR strategic decisions. Although most concepts of CSR refer to corporate behavior, it is the top decision-makers and their management teams within the enterprise who actually create, implement, maintain, or circumvent such policies and behaviors, especially the ethical leaders with distinct ethical traits in the enterprise. The fulfillment of CSR by the enterprise starts with taking effective measures to strengthen the ethical leadership of the CEO. Therefore, in the selection of managers, enterprises should not only evaluate their abilities and other leadership qualities, but also focus on their morality.

Secondly, leaders and TMTs jointly shape CSR practices and results. Ethical leaders should pay careful attention to the role of TMT members in organizational decision-making when promoting the making of CSR strategic decisions, especially proactive ones. TMT members ‘HWP will not only promote their work attitude, motivation and engagement, but also stimulate their sense of awe and responsibility for work decision-making. Ethical leaders need to share values with TMT members and carry out personalized care to promote the autonomous internalization of the motivation of TMT members, mobilize the enthusiasm of TMT members, work together to achieve common goals, and promote corporate growth.

Thirdly, enterprises need to create a relatively relaxed environment for CSR implementation. The moderating effect of performance stress indicates that the higher the performance stress is, the weaker the mediating effect of TMT HWP, the stronger the mediating effect of TMT OWP, the easier it is for enterprises to make reactive CSR strategic decisions, and the more difficult it is to make proactive CSR strategic choices. Therefore, an appropriate reduction in performance stress, especially short-term performance stress, is more helpful to address the balance between economic and social benefits as well as the long-term sustainable development of the enterprise.

### 5.3 Limitations and future directions

We must acknowledge some limitations of our studies that can be addressed by future research.

Firstly, our study relies on cross-sectional data. While we have taken measures to mitigate common method bias and established the absence of significant bias, it would enhance the rigor and robustness of our findings to incorporate longitudinal research designs or experimental methods in future studies.

Secondly, an interesting avenue for future research lies in exploring the relationship between ethical leadership, TMT, and CSR in different industries, organizational sizes, and cultural backgrounds. Additionally, the consideration of other influencing factors such as employee engagement, institutional environment, etc., can provide a more comprehensive understanding. These further studies will contribute to a better understanding of the role of ethical leadership and TMT in promoting both proactive and reactive CSR and offer valuable guidance and recommendations for the future sustainable development of organizations.

Thirdly, it would be valuable to examine the moderating effects of specific variables on the relationship between leadership and CSR. Given the historical influence of Confucianism on Chinese business ethics, exploring variables such as Confucianism, collectivism, socialist ideology, and political association could serve as relevant boundary conditions. These variables, deeply rooted in Chinese culture, may shape the leadership-CSR association in unique ways, and investigating their impact would provide a nuanced understanding of the relationship.

By considering these future research directions, scholars can expand upon our findings and contribute to a more comprehensive understanding of the dynamics between leadership, CSR, and contextual factors.

## Data availability statement

The raw data supporting the conclusions of this article will be made available by the authors, without undue reservation.

## Ethics statement

Ethical review and approval was not required for the study on human participants in accordance with the local legislation and institutional requirements. Written informed consent from the patients/participants or patients/participants legal guardian/next of kin was not required to participate in this study in accordance with the national legislation and the institutional requirements.

## Author contributions

J-jM: Conceptualization, Writing – review & editing. X-dW: Methodology, Writing – original draft. M-yX: Writing – review & editing, Supervision, Funding acquisition. Z-lH: Writing – review & editing, Data curation, Methodology. J-lY: Investigation, Writing – original draft. Y-bL: Data curation, Writing – original draft.
